# Targeting Gut Microbial Biofilms—A Key to Hinder Colon Carcinogenesis?

**DOI:** 10.3390/cancers12082272

**Published:** 2020-08-13

**Authors:** Siang-Siang Chew, Loh Teng-Hern Tan, Jodi Woan-Fei Law, Priyia Pusparajah, Bey-Hing Goh, Nurul Syakima Ab Mutalib, Learn-Han Lee

**Affiliations:** 1 Novel Bacteria and Drug Discovery (NBDD) Research Group, Microbiome and Bioresource Research Strength, Jeffrey Cheah School of Medicine and Health Sciences, Monash University Malaysia, Bandar Sunway 47500, Selangor Darul Ehsan, Malaysia; ssche40@student.monash.edu (S.-S.C.); loh.teng.hern@monash.edu (L.T.-H.T.); jodi.law1@monash.edu (J.W.-F.L.); priyia.pusparajah@monash.edu (P.P.); 2 Medical Health and Translational Research Group, Jeffrey Cheah School of Medicine and Health Sciences, Monash University Malaysia, Bandar Sunway 47500, Selangor, Malaysia; 3 College of Pharmaceutical Sciences, Zhejiang University, Hangzhou 310058, China; bey.hing.goh@monash.edu; 4 Biofunctional Molecule Exploratory (BMEX) Research Group, School of Pharmacy, Monash University Malaysia, Bandar Sunway 47500, Selangor Darul Ehsan, Malaysia; 5 UKM Medical Molecular Biology Institute (UMBI), UKM Medical Centre, Universiti Kebangsaan Malaysia, Kuala Lumpur 56000, Malaysia

**Keywords:** gut biofilm, microbiota, colorectal cancer, chemoprevention, quorum-sensing

## Abstract

Colorectal cancer (CRC) is a global public health issue which poses a substantial humanistic and economic burden on patients, healthcare systems and society. In recent years, intestinal dysbiosis has been suggested to be involved in the pathogenesis of CRC, with specific pathogens exhibiting oncogenic potentials such as *Fusobacterium nucleatum*, *Escherichia coli* and enterotoxigenic *Bacteroides fragilis* having been found to contribute to CRC development. More recently, it has been shown that initiation of CRC development by these microorganisms requires the formation of biofilms. Gut microbial biofilm forms in the inner colonic mucus layer and is composed of polymicrobial communities. Biofilm results in the redistribution of colonic epithelial cell E-cadherin, increases permeability of the gut and causes a loss of function of the intestinal barrier, all of which enhance intestinal dysbiosis. This literature review aims to compile the various strategies that target these pathogenic biofilms and could potentially play a role in the prevention of CRC. We explore the potential use of natural products, silver nanoparticles, upconverting nanoparticles, thiosalicylate complexes, anti-rheumatic agent (Auranofin), probiotics and quorum-sensing inhibitors as strategies to hinder colon carcinogenesis via targeting colon-associated biofilms.

## 1. Introduction

Colorectal cancer (CRC) is a global public health issue. According to The Global Cancer Observatory (GLOBOCAN) database, CRC is the second most diagnosed cancer among females and third among males [1]. Current statistical data show approximately 1.8 million new CRC cases were diagnosed worldwide in 2018, with 861,000 deaths which are often related to the disease only being diagnosed at advanced clinical stages [2]. These figures make CRC the third most diagnosed malignancy and second leading cause of death due to cancer globally [1]. In the United States, the American Cancer Society estimated that in 2020, there will be around 147,950 new cases of CRC and 53,200 deaths [3]. Although the overall reported incidence of CRC has been declining over the years, the numbers remain high and CRC imposes a substantial humanistic and economic burden on patients, healthcare systems and society. An alarming finding is the significant spike of CRC incidences among those below the age of 50 in the United States [4], with the same trend seen in Denmark, New Zealand, Australia, Canada and the United Kingdom [5].

The cost of CRC treatment worldwide is also escalating. Therefore, a lot of effort has been put into looking for preventive methods which are more cost-effective. There are various well-established risk factors in the development of CRC, including family history, age, gender, personal history, smoking, diet (red meat), obesity, heavy alcohol use and inflammatory bowel disease. However, recent studies have also shown a new risk factor, the formation of bacterial biofilm, which has been shown to be linked to the progression of CRC [6,7,8,9,10]. Biofilm formation is necessary for bacterial adhesion and growth; it occurs with the production of an extracellular polymer and adhesion matrix, and this causes a change in bacterial growth and gene expression. These polymicrobial biofilms act as a trigger for pro-carcinogenic inflammatory responses which eventually lead to the development of CRC [11]. Biofilm formation also reduces the bacteria’s sensitivity towards radiation and anti-bacterial agents [12,13,14].

The conventional treatments of CRC include chemotherapy and surgery, both of which are linked with significant complications. Surgery is invasive and associated with high mortality. Chemotherapeutics induce damage to DNA and initiate various signaling pathways leading to cancer cell death such as arrest of cell cycle, inhibition of DNA repair and global translation [15]. However, there are many problems with chemotherapy, including resistance to drugs, effects of cytotoxicity, and other adverse reactions. The treatment outcome also varies depending on the cancer subtype [16]. Given the high complication rate and the unpredictable response to treatment, there is a need for continuous development of better strategies for the prevention and therapy of CRC; targeting microbial biofilm could be a useful adjuvant strategy supporting the existing chemotherapy regimens for CRC by limiting their adverse effects, or by enhancing their efficacy. In this review, we will discuss and summarize the significance of gut-microbial biofilms and their role in colon carcinogenesis as well as explore the various strategies that could hinder the formation of biofilms and potentially prevent CRC, such as the use of natural extracts, probiotics, quorum-sensing inhibitors, anti-rheumatic agents (Auranofin), silver nanoparticles, upconverting nanoparticles and thiosalicylate complexes.

## 2. Research Methodology

The research methods were focused on locating primary research papers which investigated the potential link between colonic biofilms and colon carcinogenesis, and the various antibiofilm strategies that can target gut microbial biofilms of *B. fragilis, E. coli and F. nucleatum.* A systematic search using Google Scholar, Ovid Medline and Pubmed was done to identify published articles on the subjects above. Keywords “colorectal cancer”, “biofilms” and “antibiofilm” were the keywords used to search for relevant articles. Other supplemental keywords including “mechanism of action”, “*Bacteroides fragilis*”, “*Escherichia coli*” and “*Fusobacterium nucleatum*” were combined with “antibiofilm” using Boolean operators.

## 3. The Role of Colonic Microbiome and Biofilm in Colon Carcinogenesis

Over the past 20 years, extensive research on the human microbiome has indicated that human health, while heavily related to our own genome, is linked to a great degree on microbes which are living in and on our body [17,18,19,20,21]. The term microbiota refers to the collection of microorganisms present in a defined environment, including fungi, viruses and bacteria. It was defined by Lederberg and McCray [22] who highlighted the importance of microorganisms colonizing the human body in the implications for health and disease. Microbiome generally refers to the entire habitat which includes the microorganisms, their genomes and genes and the surrounding environmental conditions [23]. In the human body, the gastrointestinal tract is the site most densely populated with microorganisms—hosting about 40 trillion microbes constituting more than 1000 species, the majority of which inhabit the colon [24]. Given that they represent the largest surface area for the interactions between the host immune system and colonic microbiota, these microorganisms are expected to exert a profound influence on human physiology and metabolism. Thus, it is unsurprising that a shift of gut commensal microbiota towards opportunistic pathogens will negatively impact the physiological functions and serve as a primary driver for intestinal inflammation which increases the risk of CRC [25,26].

**Figure 1:**
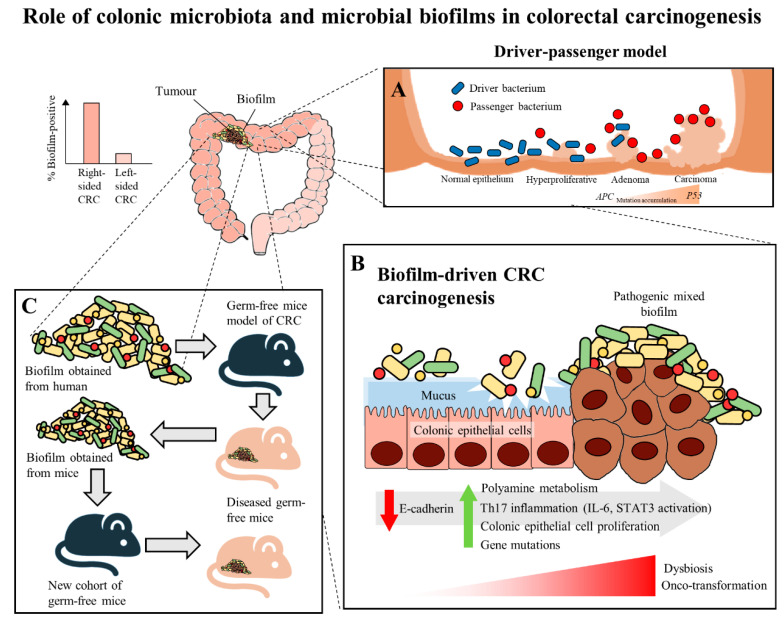
The role of colonic microbiota and biofilm in colorectal cancer (CRC) carcinogenesis. (**A**) The driver-passenger model for CRC carcinogenesis [39]. (**B**) Biofilm-driven CRC carcinogenesis. Biofilm results in loss of colonic epithelial cell E-cadherin (consistent with disrupted intestinal barrier function), increased IL-6 expression and STAT3 activation. These microbial biofilms contribute to a pro-oncogenic and pro-inflammatory state, coupled with the increased polyamine metabolism in colonic tissues, hence resulting in dysbiosis and onco-transformation and leading to tumor progression [11]. (**C**) The reassociation experiment showing that the microbiota communities from human biofilm-positive mucosa (healthy or CRC patients) resulted in CRC development in a new cohort of mice, indicates these biofilm-positive microbiota communities maintained their tumorigenic capacity [6].

In recent years, multiple studies have shown that there are specific microorganisms which are associated with the development of CRC, and these microorganisms play a role in inducing tumorigenesis in genetically susceptible murine models of disease [27,28,29]. These results further led to the identification of microorganisms which harbor pro-oncogenic genes associated with CRC, which includes *Fusobacterium nucleatum* through its expression of FadA and Fap2 adhesins [30], *Escherichia coli* through its virulence factors that allows it to harbour genomic *pks* islands [31] and enterotoxigenic *Bacteroides fragilis* through its expression of the *B. fragilis* toxin (BFT) [32]. In the development of CRC, it was proposed that the keystone pathogens, such as *B. fragilis*, act as the main drivers in CRC initiation via their direct genotoxic effects, leading to a T helper type 17 (Th17) inflammatory response in the colon [32]. The recruitment of immune cells releases genotoxic oxygen radicals which can cause multiple DNA double-strand breaks, resulting in an inflammation-driven carcinogenesis [33,34,35]. This would also lead to an increased proliferation of intestinal epithelium due to the activation of proto-oncogenes and mutations of tumor-suppressor genes. As a result, the cumulative effects of the sustained inflammation and epithelial hyperplasia together with host genetic factors associated with CRC susceptibility further drive the initiation of CRC [36]. Moreover, the Th17-dependent inflammation induced by the driver pathogens may alter the tumor microenvironment and generate novel ecological niches for opportunistic pathogens (passenger pathogens) which eventually outcompetes the driver bacteria during CRC progression. Tjalsma, et al. [37] described this process as the “bacterial driver-passenger model”, thereby the passenger pathogens such as *Fusobacterium* spp. or *Streptococcus* spp. gradually colonize the colonic mucosa leading to intestinal microbial dysbiosis and causing CRC progression (Figure 1A). Although current evidence has shown that *F. nucleatum* appears to be actively involved in the later stages of CRC progression [6,30], the definite role of *F. nucleatum* as a passenger or a driver is still elusive. Earlier, Kostic, et al. [38] showed that *F. nucleatum* plays a vital role as a driver capable of promoting CRC progression, where the mutated adenomatous polyposis coli (*APC*) gene is required for *F. nucleatum* in inducing CRC progression in a mice model.

The human colon is surrounded completely by a protective mucus barrier comprised largely of mucins, particularly, mucin 2 (MUC2), which prevent the colonic epithelium of the host from coming into direct contact with the microbiota. The mucus barrier is organized in two layers. The inner, stratified mucus layer adheres firmly to the epithelial cells. It is dense and does not allow penetration of bacteria. Thus, it functions to separate the commensal bacteria from the host epithelium while the outer, non-attached layer acts as the natural habitat for the commensal bacteria. This mucus barrier allows normal intestinal microbiota to inhabit the colonic mucus without activating an inflammatory response [39]. When there is a breach in the protective mucus barrier, this allows the microbiota to come into contact with the colonic epithelium and this has been suggested to be an important step that initiates modifications in the epithelial cells causing intestinal inflammation [40]. The increased access to the colonic epithelium causes modification of the microbial community relationships, thereby changing the microbial composition and activity, often resulting in the formation of a biofilm [39,41]. Biofilms refer to polymicrobial communities which are enclosed in a matrix that forms on abiotic and biotic surfaces. It starts with microcolonies (small aggregation of bacterial cells) attaching to the surfaces and these adherent microcolonies then form mature biofilms when they become encapsulated in a matrix composed of self-secreted polysaccharides [42]. Having the ability to form biofilms, confers to these polymicrobial communities increased tolerance to antibacterial drugs and immune clearance [43]. Nutrients and water are also held by the embedded bacterial communities in the matrix of the biofilm [44]. Hence, biofilms favor the survival and persistence of the polymicrobial communities.

Recently, biofilms have been associated with the onset and progression of CRC, a feature particularly evident in the proximal colon up to the hepatic flexure (right-sided CRC). The occurrence of biofilms is more frequently seen in colonic tissue samples from CRC patients compared to healthy individuals [41]. There is a theory that the biofilms harbor different bacterial species, rather than a single solitary variant of the invading microorganism, and possibly cause increased inflammatory responses and production of bacterial-derived genotoxic compounds. Studies by Drewes, et al. [45] and Dejea, et al. [41] have revealed that the majority of sporadic CRC patients with colonic tumors proximal to the hepatic flexure harbored mucosal biofilms while only a small portion of CRC patients with tumors distal to the hepatic flexure had biofilms. These observations may explain the poorer prognosis of right-sided CRC [46] as the biofilm-positive CRC may have additional serious epithelial tissue injury and intestinal inflammation [11]. Despite that, mucosal biofilms were also found in about 13% of healthy individuals who underwent routine screening colonoscopy. However, these studies did not investigate the cause-and-effect relationship between cancer-associated biofilms and CRC carcinogenesis, but merely demonstrated a novel and compelling perspective in illustrating the involvement of microbial biofilms as a holistic entity rather than proving a causal association of a specific microbial pathogen [47].

There have been numerous mechanisms proposed to illustrate the role of mucosal biofilms in mediating the process of CRC carcinogenesis (Figure 1B). In either healthy individuals or CRC patients, the gut microbial biofilm communities are consistent with pro-oncogenic biological changes: there is an increased proliferation of colon epithelium, increased IL-6, STAT3 activation, increased synthesis of polyamine and reduction of E-cadherin [9,41]. The increased levels of polyamine metabolites were suggested to act synergistically to promote biofilm formation and cellular proliferation, creating conditions conducive to oncogenic transformation in colon cells [9]. Moreover, the changes in permeability of the colonic barrier and metabolism of cells causes the microenvironment of the tumor to change in such a way that these initial pathogenic bacteria drivers gradually get replaced by tumor-foraging opportunistic bacteria pathogens with a competitive advantage in the tumor niche [37].

On the other hand, biofilms have also been detected in familial adenomatous polyposis patients who have inherited a mutation in the *APC* gene and are highly prone to CRC due to the development of polyps and adenoma formation as the early stage of the “adenoma-carcinoma sequence” [8]. The “adenoma-carcinoma sequence” model was developed by Fearon and Vogelstein [48], which is a multistep process that illustrates the accumulation of genetic and epigenetic mutations as the drivers for the onset and progression of CRC. Briefly, the sequence usually begins with the *APC* gene mutation, and ends with the *P53* mutation, after which it progresses into carcinoma. Son, et al. [49] observed that the gut microbial composition is altered in *Apc^Min^* mice prior to obvious outgrowth of intestinal polyps. The *Apc^Min/+^* mice are often used to study human colon carcinogenesis, as the mice harbor a truncated *APC* gene and develop multiple intestinal neoplasia (Min) [50]. Therefore, Li, et al. [11] suggested that the microbial biofilm may be regarded as the driver in the adenoma–carcinoma sequence at an early stage of CRC progression.

Moreover, studies show that the commensal (*Parvimonas*, *Peptostreptococcus*, *Prevotella*) and the pathogenic (*F. nucleatum*, *P. gingivalis*) periodontal bacteria, which are capable of forming biofilms, are detected in the intestinal biofilms [45,51]. Thus, an interesting hypothesis was proposed to illustrate the potential involvement of oral microbiota in CRC development, whereby the oral periodontopathic bacteria may have translocated into the colorectum, contributing to intestinal dysbiosis. This presents a new outlook on CRC pathogenesis which is driven by the orally-derived colonic biofilm [52].

Intriguingly, a recent murine study by Tomkovich, et al. [6], intended to delineate the causality of microbial biofilms in CRC, successfully demonstrated that the polymicrobial biofilms are carcinogenic in a preclinical in vivo experiment with the use of three genetic murine models of CRC carcinogenesis (germ-free *Apc^Min^*^Δ*850/+*^; *Il10^−/−^* or *Apc^Min^*^Δ*850/+*^ and specific pathogen-free *Apc^Min^*^Δ*716/+*^ mice). The study showed that at 12 weeks after inoculation, inocula prepared from human colon mucosa covered with biofilm induced the formation of colon tumors, primarily in the distal colon; while no colon tumors were induced by the inocula prepared from the biofilm-negative colon mucosa. Furthermore, within the first week after inoculation, the biofilm-positive human tumor homogenates, which were not seen in biopsies of healthy individuals, showed consistent invasion of the mucosal layer and formation of biofilm in mouse colons. A remarkable finding of this study was that biofilm communities from the colon biopsies of healthy individuals were as potent as biofilm communities from CRC hosts in inducing development of tumors. This finding is pertinent as the presence of polymicrobial biofilms containing the potential pathogens present an increased risk for CRC development and is regarded as a tipping point between a healthy and a diseased gut mucosa [42]. Furthermore, the latest finding also showed that similar levels of inflammation were observed in both mice inoculated with biofilm-positive and biofilm-negative control homogenates, but a lower degree of immunosuppressive myeloid cell recruitment and IL-17 production was triggered by biofilm-negative control homogenates when compared to biofilm-positive homogenates in the mice [6]. Often, infiltration by immune cells is associated with adverse clinical outcomes of CRC [53]. This shows that the colonic biofilms interact and alter the mucosal immune responses, possibly via the Th17 pathways, thereby promoting CRC carcinogenesis [6].

The carcinogenicity of biofilm-positive human tumor microbiota were further reinforced via the reassociation experiment showing the development of colon tumors in a new cohort of mice after inoculation with homogenized proximal or distal colon tissues from biofilm-positive inoculated mice (Figure 1C). This finding shows that the microbiota communities from human biofilm-positive mucosa which assembled in the first group of germ free mice maintain their tumorigenic capacity, even after being transferred to the new group of germ free mice [6]. Metatranscriptome analysis revealed differential upregulation of microbial genes which are involved in bacterial invasion of epithelial cells and the biosynthesis of peptidoglycan in the mucosa of mice associated with a biofilm-positive tumor compared to biofilm-negative biopsies [6]. Taken together, all these findings further fortify the notion that the formation of a biofilm by microbial pathogens appears to play a vital role in the induction and the progression of CRC.

## 4. Strategies to Target Gut Microbial Biofilms

As gut microbial biofilms play such an important role in colon carcinogenesis, preventative approaches aimed at the detection and eradication of bacterial biofilms might be beneficial for individuals at risk of CRC. The formation of a biofilm results in the increased resistance of the bacteria to antibiotics and antimicrobial agents. To date, there is no antibiotic that has been proven effective in treating biofilm related infections due to their larger values of minimum inhibitory concentration (MIC) and minimum bactericidal concentration, which may cause in-vivo toxicity. Studies on antibiotics, such as colistin and imipenem, among others, have shown that they are unable to eliminate a biofilm entirely, merely reducing it [54,55,56,57]. Recent studies also demonstrated that the use of broad-spectrum antibiotics was not able to produce favorable clinical outcomes in patients with various types of cancer including CRC [58]. Biofilms result in the weakened activation of phagocytes and the complement system, thus protecting pathogenic bacteria from the host’s immune system and contributing to the increased resistance of up to about 1000-fold against conventional antibiotics [54,59]. The structure and nature of biofilm, the availability of oxygen and nutrients to the bacterial cells as well as acquired and intrinsic bacterial resistance are the other factors contributing to the increased tolerance to antimicrobial actions [43]. This was further reinforced by a study on *P. aeruginosa* whereby the mucoid nature of the biofilm confers resistance towards tobramycin [60]. The metabolic state of the bacteria in the biofilm and limited oxygen supply are also possible factors that contribute to its resistance towards antimicrobial agents [61]. Jeyaraj, et al. [62] found that when antibiotics are given at sublethal concentration, biofilm cells mutate at a higher rate than their planktonic counterparts, thereby increasing the chances of antibiotic resistance gene transfer via plasmids. Therefore, we are exploring various antibiofilm strategies that could potentially be new chemopreventive agents and adjuvants against CRC by targeting gut microbial biofilms. These strategies are summarized in Table 1.

### 4.1. Natural Products

Natural products have long been a “gold mine” for therapeutic entities that exhibit a myriad of biological activities [79,80,81]; with many natural products having been shown to possess promising antimicrobial and antibiofilm activities [82,83]. Considering the vast diversity in chemical structures and their known bioactivities, natural products derived from plants have emerged as attractive candidates for drug development to suppress biofilm formation as well as eradicate biofilms formed by pathogens [84,85]. In this review, several plant-derived natural products recently reported to exhibit antibiofilm against the enteropathogens associated with CRC are highlighted as below.

Enterotoxigenic *B. fragilis* has been suggested to be a keystone pathogen in the initiation of colon carcinogenesis [86]. Other bacteria associated with CRC progression include *F. nucleatum* and *E. coli* [11]. There are a few studies of natural extracts which have shown antibiofilm effects towards biofilms containing such pathogens and could potentially be explored further in more clinical trials as alternatives for CRC prevention [87]. One of the strategies is the use of zerumbone extracted from *Zingiber Zerumbet* (L.) Smith which is a type of edible ginger. In the past few years, studies have suggested that zerumbone has many biological activities which promote anti-mutagenic, anti-bacterial, anti-cancer and anti-inflammatory activities [88,89,90]. Recently, Kim, et al. [63] demonstrated that zerumbone exerted antibiofilm activities against different strains of *B. fragilis*, including the wild-type enterotoxigenic *B. fragilis* (WT-ETBF), *btf-2* gene overexpressing ETBF and non-enterotoxigenic *B. fragilis*. Zerumbone was shown to inhibit biofilm formation as well as eradicate the preformed biofilm. Interestingly, the study demonstrated that zerumbone inhibited biofilm formation of *B. fragilis* strains containing the toxic *bft-2* gene more effectively than the non-enterotoxigenic strain. Furthermore, the study suggested that the antibiofilm activity of zerumbone may be mediated via the downregulation of an efflux pump-related gene (*bmeB12*) which has been associated with biofilm formation [63]. Alpha-humulene is another natural product showing potential to inhibit biofilm formation by enterotoxigenic *B. fragilis* [64]. Alpha-humulene is a sesquiterpene found in the essential oils of aromatic plants, including *Mentha spicata*, *Salvia officinalis* and ginger family (Zingiberaceae) [91,92,93]. Alpha-humulene has been known for its anti-inflammatory actions and a few studies have shown that essential oils with α-humulene have antibacterial effects [94,95]. Similar to zerumbone, α-humulene was also shown to exert antibiofilm activity by inducing downregulation of RND-type efflux pump *bmeB1* and *bmeB3* genes, leading to cell membrane disruption and the suppression of biofilm formation of enterotoxigenic *B. fragilis* [64].

Antibiofilm effects towards biofilms produced by *E. coli* were seen in a study of *Sauropus androgynus* leaf extracts whereby both antibiofilm and antimicrobial activity were demonstrated. Through gas chromatography-mass spectrometry (GC-MS) analysis, these leaf extracts were found to contain phytochemicals, such as steroids, phenols, alkaloids, tannins and flavonoids, which possess antimicrobial, anti-inflammatory, anticancer and immunomodulatory properties [96,97,98]. Bazargani and Rohloff [65] suggested coriander essential oil as a new antibiofilm agent. They studied the antibiofilm activity of essential oils and plant extracts of anise, coriander and peppermint against *E. coli*. Their study showed that coriander essential oil had the highest antibiofilm activity against the biofilm produced by *E. coli*. Previous studies have shown that terpenes have antimicrobial activity due to their ability to modify the permeability of cells by the penetration of membrane lipid bilayers through fatty acyl chains, distortion of lipid packing and alteration of the cell membrane’s fluidity [65,99,100]. The presence of terpenes, such as *p*-cymene, octanol, geranyl acetate, α-pinene, γ-terpinene and linalool, could also be the reason coriander essential oils have a high inhibitory effect on formation of *E. coli* biofilms [65]. In Asian countries, pomegranate has been used in traditional medicine for treating diarrhea and dysentery for many years. There were multiple reports on the bioactive potential of pomegranate as an antibacterial, antioxidant and anticancer agent [101,102,103]. One of the studies indicated that the pomegranate extract and ellagic acid, which is its major component, exhibit antibiofilm activity against *E. coli* [66].

### 4.2. Anti-Rheumatic Agent

Auranofin is a gold salt and has been approved by the Food and Drug Administration (FDA) as a drug to treat rheumatoid arthritis. It has been proposed to be repurposed as an antibacterial and antibiofilm agent against intestinal bacteria, such as enterotoxigenic *B. fragilis*, and potentially as an anti-cancer drug. Repurposing of auranofin would be cost and time efficient as it saves the time and expense needed to develop and test a new drug; given that it is already approved and has been in use for several years, its safety has already been extensively studied [73]. A study in 2014 showed that auranofin displayed antitumor activity against a p53-null ovarian carcinoma SKOV3 cell line [104]. Other studies showed its antibiofilm and antibacterial actions against methicillin-resistant *Staphylococcus aureus* (MRSA) and vancomycin-resistant enterococci (VRE) [105,106]. This sparked interest among Jang and Eom [73] to investigate the antibacterial and antibiofilm effects of auranofin against enterotoxigenic *B. fragilis*. Their study demonstrated promising results of auranofin against enterotoxigenic *B. fragilis* with relatively low concentrations required to inhibit and eradicate both the biofilms and bacteria. Treatment with auranofin was shown to induce significant reduction of expression of the outer membrane protein (*ompA*) gene and the *bmeB3* gene [73]; the *ompA* gene has been associated with the regulation of biofilm formation [107]. Future studies should be conducted to determine the efficacy of Auranofin to inhibit enterotoxigenic *B. fragilis* in in vivo models.

### 4.3. Probiotics

The Food and Agriculture Organization of the United Nations and the World Health Organization (FAO/WHO) have defined probiotics as “Live microorganisms which when administered in adequate amounts confer a health benefit on the host” [108]. Probiotics are regarded as non-pathogenic and safe, and are commonly used as live supplements for their health promoting effect via maintaining intestinal microbial balance [109]. Probiotic strains have been used to treat microbial infections, boost human health and have displayed promising use in preventing antibiotic associated diarrhea, necrotizing enterocolitis in preterm infants, as well as in treating infantile colic, periodontal disease and inducing or maintaining remission of ulcerative colitis [110,111,112,113,114].

Based on the current evidence, there are examples of probiotics showing promising results in controlling biofilm formation by pathogens which cause biofilm-associated diseases at different sites of the host, including the oral cavity [115], wounds [116] and the gastrointestinal tract [117]. Several preclinical experiments demonstrated that probiotics and their derived products can be potentially developed to target carcinogenic biofilms. In these studies, antibiofilm properties of cocktails of different probiotic strains were evaluated against the biofilm-growing enteropathogens, including enterotoxigenic *B. fragilis* and enterotoxigenic *E. coli* strains [74,75]. The effects of the probiotic *Clostridium butyricum* NCTC 7423 supernatant on gene expression and formation of biofilm of enterotoxigenic *B. fragilis* was recently studied by Shin, et al. [74]. The cell-free supernatants (CFS) of *C. butyricus* exhibited antagonistic effects against the growth of enterotoxigenic *B. fragilis* in planktonic culture. CFS from *C. butyricus* also inhibited the development of biofilms, dissembled biofilms which were preformed and decreased the metabolic activity of cells in the biofilms. It was also shown to significantly reduce the expression of virulence and efflux pump related genes in enterotoxigenic *B. fragilis,* such as *bmeB3* and *ompA*. In addition, CFS from *C. butyricus* showed the ability to significantly suppress extracellular nucleic acids and proteins within the basic components of the biofilm [74]. Furthermore, another study demonstrated a preparation of cell-free spent media (CFSM) of six probiotics which belong to the genus *Bifidobacterium* and *Lactobacillus* exhibited strong antibacterial activity against all *E. coli* isolates and were able to suppress growth of drug-resistant *E. coli*. The CFSM of probiotics in this study were also able to reduce the formation of biofilm of two multi-drug resistant *E. coli* [75].

**Figure 2:**
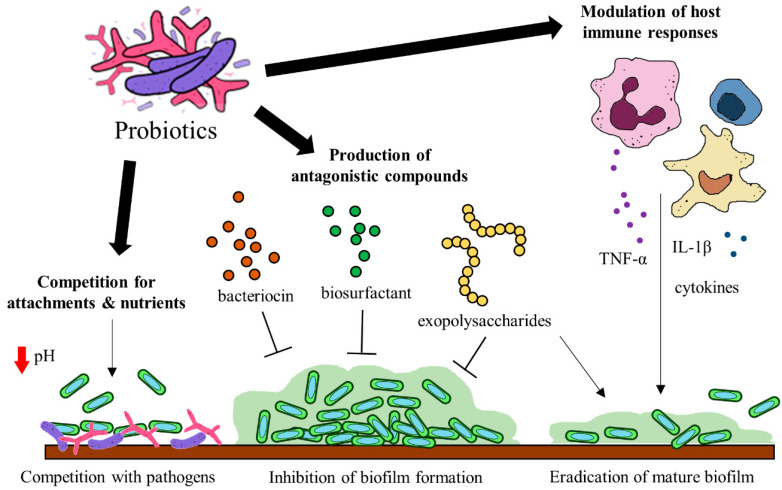
Potential mechanisms of probiotics to target gut microbial biofilms.

Probiotics have been identified to hinder biofilm formation and the survival of biofilm pathogens with different mechanisms. Some of these mechanisms include (a) the production of antagonistic compounds, (b) competition with pathogens and (d) modulation of host immune responses [118] (Figure 2). Probiotics can produce various antagonistic compounds, including exopolysaccharides [119], bacteriocins [120] and biosurfactants [121] which exhibit antibiofilm activity. These antagonistic compounds have been shown to interfere with biofilm attachment and formation as well as the thinning of mature biofilms. Furthermore, probiotics are capable of competing with the pathogenic bacteria for surface of attachment and nutrients by altering their surrounding pH values [122,123]. Besides the direct interactions between probiotics and the pathogens, probiotics exert immunomodulatory effects via interaction with the immune system when administered into a host. Studies suggest that probiotics and their soluble factors can regulate and activate specific immune cells and the release of cytokines via toll-like receptor recognition to elicit immunomodulatory effects [124,125].

Although promising preclinical results have been demonstrated, there is still insufficient evidence to consider probiotics as a strategy to prevent the onset or progression of CRC by means of inhibiting pathogenic biofilm formation or disrupting the pre-formed biofilms. Future studies should elucidate the molecular mechanism of probiotic action in the gut of well-designed animal models or clinical studies related to biofilm-associated CRC to provide a clearer picture of how probiotics act on the bacterial communities in biofilms and contribute to the prevention of CRC initiation and progression. At present, the intake of probiotics has shown promising results in several clinical trials and has been suggested as a viable chemopreventive approach to combat colorectal carcinogenesis via modulation of gut microbiota [126,127,128]. Based on this evidence, we can envisage that probiotic interventions represent an alternative strategy or adjuvant in the treatment of biofilm-associated diseases.

### 4.4. Quorum Sensing Inhibitors

Bacterial quorum sensing (QS) plays an important role in the formation of microbial biofilms. QS is a type of population density-dependent cell-to-cell communication where it activates specific signals to coordinate pathogenic behaviors and helps bacteria adapt to undesirable growth conditions [76]. It plays a significant role in regulating expression and transfer of virulence-associated bacterial genes [77]. The QS signals in the bacteria consist primarily of autoinducing peptide, acyl-homoserine lactone (AHL) and autoinducer-2 [76]. QS regulates bacterial active efflux pumps, which can discharge antibiotics from the bacteria effectively, hence playing a role in promoting multidrug resistance. The QS system also plays a regulatory role in biofilm formation of Gram-positive and Gram-negative bacteria. Our concern is with Gram-negative bacteria. The formation of biofilm of Gram-negative bacteria is controlled by the QS system using the AHL signal molecule, which consists of signal molecules and the corresponding receptors. As the density of bacteria increases, the secretion of signal molecules also increases and once the signal molecules reach a definite threshold, they bind to and activate the corresponding signal molecule receptors. Once activated, the receptors trigger the relevant transcriptional regulators to produce extracellular polysaccharides, alginates and toxin factors which promotes the formation of biofilms [78]. QS inhibitors have provided new possibilities for overcoming microbial resistance and biofilm formation. QS inhibitors can work in three main ways: inhibition of the synthesis of signal molecules, degradation of QS signals or interference with signal reception for QS blockage [76,78]. There are a number of small-molecules have been identified to be effective in inhibiting the QS system of human pathogens, including flavonoids (apigenin, baicalein, quercetin) [129], *N*-decanoyl-L-homoserine benzyl ester [130] and meta-bromo-thiolactone [131]. For instance, flavonoids were demonstrated as inhibitors of both QS receptor LasR and RhlR, resulting in repression of biofilm formation in *Pseudomonas aeruginosa* [129]. The genus *Pseudomonas* has also been shown to be a potentially opportunistic pathogen which might increase the risk of colorectal adenoma development [132]. Methylthio-DADMe-immucillin-A is another example of a QS inhibitor that has been studied for its disruption of QS of *E. coli,* and it does so by the inhibition of signal synthesis, suggesting a promising strategy for targeting biofilms associated with CRC progression [133].

### 4.5. Silver Nanoparticles

In recent years, research has been focused on studying metal-based nanoparticles and their use in targeting and treating many health diseases including secondary infections and cancer [134]. Silver nanoparticles (AgNPs), particularly, have been researched extensively for their use in different fields including food packaging, environment and healthcare [135]. The cytotoxic potential of AgNPs were demonstrated on a few cancer cell lines including cervical cancer (HeLa), colon cancer (HT29), breast cancer (MCF-7) and lung cancer (A549) [62,136,137,138]. Besides, AgNPs also exhibited antimicrobial potential through the release of silver ions which are biologically active when silver is ionized in aqueous solution [139,140]. Since then, there have been various studies focusing on developing silver nanoparticles that could display bactericidal and antibiofilm actions. Several studies have adopted the emerging green synthesis approach by synthesizing AgNPs from plants, providing an inexpensive, efficient and eco-friendly alternative to the conventional NPs synthesis [67,141]. One of these studies demonstrated that biosynthesized AgNP from AgNO_3_ and *Pandanus odorifer* leaf extract using microwave irradiation exhibited antimicrobial and antibiofilm activities against *E. coli*. The production of exopolysaccharides and swarming mobility, which are important factors necessary in the initial attachment and maturation of biofilm, were significantly decreased upon exposure to AgNPs. Another showed that the AgNP which was green synthesized from *Gloriosa superba* aqueous leaf extract exhibited antibiofilm activity against *E. coli* [68]. Furthermore, an in vivo study indicated that the toxicity of biosynthesized AgNPs was mild, with minor effects on the liver and renal functions of the mice; nevertheless more studies should be conducted to substantiate its therapeutic use in treating biofilm-associated CRC [67].

### 4.6. Upconverting Nanoparticle

Upconverting nanoparticles (UCNPs) are a special class of photoluminescent materials which are able to exploit the up conversion of photons [142]. UCNPs are lanthanide-doped nanocrystals that are triggered by light which have been proposed to be used to detect and treat CRC. UCNPs transform long-wavelength near infrared (NIR) excitation light into emissions with short wavelength. This allows light to penetrate deeper and have a high signal-to-noise ratio. Recent in vitro and preclinical studies indicate that with various modifications, UCNPs can pick up bacterial infection and inflammation, which usually precedes CRC. UCNPs are able to detect specific pathogens which are responsible for development of CRC [69]. For example, UCNPs coupled with an anti-*Escherichia coli* antibody are able to detect *E. coli * [69,70]. Besides use for bacterial detection, UCNPs also have antimicrobial and antibiofilm applications. When UCNPs are used as a core covered with a shell surface made of TiO_2_ modified with *d*-amino acids, the UV light can stimulate the outer TiO_2_ shell to form reactive oxygen species which have antibacterial actions and stimulates the release of free *d*-amino acids which have antibiofilm properties [69,71].

### 4.7. Thiosalicylate Complexes

Thiosalicylate complexes of Zn(II) and Hg(II) are proposed to be a new class of antibiofilm, antimicrobial compound with anti-tumor effects. Thiosalicylate complexes of Zn(II) and Hg(II), [Zn(SC_6_H_4_CO_2_)(TMEDA)]_2_, were shown to have potent antimicrobial and antibiofilm activities against *E. coli*, whereby complete degradation of *E. coli* biofilms was achieved at relatively small concentration of 0.227 μg/mL. Hence, the study suggested that the complexes hold great promise for the development of a new class of antibacterial and antibiofilm agents to combat the resistant pathogens [72]. Thiosalicylate complexes of Zn(II) and Hg(II), [Zn(SC_6_H_4_CO_2_)(TMEDA)]_2_, were also shown to exert anti-tumor actions against the colon cancer cell line HCT116 [72]. Thus, it is worthwhile exploring the clinical use of thiosalicylate complexes as promising agents for preventing CRC.

## 5. Discussion

In the recent decade, much evidence has shown that microorganisms are involved in the progression of CRC. Studies show an association between colon carcinogenesis and biofilm formation by cancer-associated bacteria, thus presenting unprecedented opportunities to develop potential chemopreventive strategies for CRC by targeting these microbial biofilms.

The early and accurate identification of adenomatous colonic polyps in high-risk patients is crucial to prevent CRC progression and enhance the chances of a successful treatment by the removal of the adenomatous polyps. The presence of bacterial biofilms composed of *E. coli* and enterotoxigenic *B. fragilis* on the colonic mucosa of patients with familial adenomatous polyposis (benign precursor lesions) was suggested to accelerate the colon carcinogenesis [8]. In addition, Dejea, et al. [41] demonstrated that the patients with biofilms are more likely to develop CRC than those without biofilms. Thus, the development of a minimally invasive approach to detect the presence of these biofilms in patients with high risk of developing CRC could be a useful screening and preventive tool for CRC. Due to the lack of sensitivity and sufficient invasiveness from the conventional imaging techniques, including endoscopy which has a high miss rate that varies from 6% to 27% especially of the flat and depressed neoplasms [143,144], UCNPs emerge as the better bioimaging strategy with increased detection sensitivity, deeper tissue penetration and less non-specific tissue autofluorescence. In addition, UCNPs can be a promising bioimaging strategy not only to detect the precancerous polyps, but they have also been shown to be effective in detecting the presence of biofilms containing the enterotoxigenic pathogens and even exert antimicrobial and antibiofilm actions when coupled with antimicrobial agents [69,71]. Collectively, these studies highlight the potential use of UCNPs as new treatment strategies in CRC.

Although NPs are widely used in various fields such as biomedicine, agricultural and industrial sectors, the broad applications of NPs have raised great concern regarding their possible effects on human health and the environment [145]. There is evidence that ingestion of NPs alters the intestinal microbiota composition in favor of pathogenic species, causing deleterious effects on beneficial bacteria. For the case of AgNPs, a number of studies indicated that AgNPs could promote adverse consequences on human gut microbiota, with the evidence of perturbations in bacterial composition, and the potential alternation of mucosal immune responses which are related to colitis [146,147,148]. Likewise, long-term oral exposure of TiO_2_ nanoparticles (NPs) has been shown to elicit and exacerbate intestinal inflammation in mice [149,150]. Hence, the long-term impact of TiO_2_ NPs on humans requires extensive investigations prior to clinical use and the use of TiO_2_NPs should be done with caution, particularly in patients with pre-existing inflammatory conditions [150]. Given that most of the toxicity studies were performed with high doses causing acute toxicity, it has been suggested that future studies should be performed at relevant doses to further delineate the interactions between the NPs and gut dysbiosis as well as chronic effects in human health. Nevertheless, these NPs and nanocomposites may not act solely but in mixtures making their interactions and effects difficult to be properly interpreted in a biological system [145].

Natural products offer a great resource with undeniably diverse chemotypes and pharmacological activities [151,152,153]. Epidemiological and experimental studies indicate an association between dietary components, gut microbiota and colorectal cancers, thereby the natural products present in our diet are able to confer protection against colorectal cancer via the modulation of gut microbiota [154,155,156]. As indicated earlier in this review, the natural dietary plant metabolites such as zerumbone, α-humulene, coriander essential oil and pomegranate extracts are suggested to be promising substances for prevention of colon cancer by targeting the biofilm-producing gut pathogens associated to cancer. However, most the studies demonstrated the in vitro antibiofilm efficacy of the bioactive natural products against the cancer-associated pathogens. On top of that, it is also important to study the effects of these natural products on a complex mixed biofilm community: not only its impact on a specific gut pathogen but also its potential effect on the complex commensal community. In fact, as aforementioned the recent evidence revealed that the colonic biofilms are composed of polymicrobial communities and have been shown to be carcinogenic [5]. Furthermore, data from the in vivo studies are extremely critical to determine the clinical utility of these natural products in treatment of CRC. Nonetheless, there is much in vitro and in vivo evidence on the chemotherapeutic and chemopreventive effects of these natural products against various cancers, including CRC [89,157,158]. Thus, future research could investigate the modulatory effects of these natural products on biofilm of the complex microbial communities present in the intestinal tract yet correlating with its anticancer effect in colon carcinogenesis animal models.

Undeniably, one of the greatest challenges to target or to treat disease affecting the colon is the fact that the colon is located at the distal part of the gastrointestinal tract. Hence, the development of a colon-specific therapy is commonly associated with challenges with respect to the physiological complexity in the gastrointestinal tract, the intrinsic properties (colonic bioavailability) and the specific site targeting abilities of the interventions. To improve the success of a colon-specific targeting strategy, there are different colon-targeted drug delivery systems have been devised over the years, primarily to preserve the formulation during its passage through the stomach and the small intestine, and eventually reach its target site [159,160]. The use of nanoparticles with inherently antimicrobial activity, such as silver nanoparticles, to treat biofilms has been extensively explored [161,162], while the study of nanoparticles to deliver small molecules or quorum sensing inhibitors to biofilms is relatively limited. Nanoparticles represent an ideal vehicle for sustained and controlled release of the antibiofilm agents as they are capable of protecting the small molecules from enzymatic degradation and prevent electrostatic binding to the extracellular polymeric substance produced by the biofilms, thus rendering enhanced antibiofilm efficacy [161,163]. Besides that, chitosan and poly(lactic-co-glycolic acid) nanoparticles are examples of other classes of nanoparticles worthy to be mentioned and deserve more future investigations on their feasibility as a promising strategy for the targeted delivery of drugs to the colon [163].

## 6. Conclusions

The formation of biofilms has been suggested to play a role in the initiation of colon carcinogenesis, and hence the inhibition or removal of such biofilms could represent a promising strategy for CRC prevention and treatment. The current research focus on biofilm inhibitors and quorum sensing inhibitors against biofilms of enterotoxigenic *B. fragilis* or *E. coli* are valuable for the future development of new drugs. Most of these studies produce promising in vitro results. As of yet, limited in vivo evidence is available and more in vivo studies are needed to further explore the potential of natural products, the anti-rheumatic agent auranofin, probiotics, quorum-sensing inhibitors, silver nanoparticles, UCNPs and thiosalicylate complexes in the prevention and treatment of CRC. It will also be worthwhile looking into methods of improving the application of those strategies in terms of their methods of delivery to the colon by modifying them and employing colon-targeted drug delivery systems to enhance their ability to target gut microbial biofilms. There should also be continuous efforts to invent new formulation technologies that can improve colon-targeted drug delivery systems.

## Figures and Tables

**Table 1:** Strategies to target gut microbial biofilms.

Antibiofilm Strategies	Antibiofilm Agents	Findings		References
Natural Products	Zerumbone	Antimicrobial activity against *B. fragilis * Inhibit formation of *B. fragilis* biofilms Complete eradication of *B. fragilis* biofilms Decreases expression of bmeB12	MIC: 32 μg/mL	[63]
	Alpha-humulene	Antimicrobial activity against *B. fragilis * Inhibit formation and eradication Complete eradication of *B. fragilis* biofilms	MIC: 29 μg/mL	[64]
	Coriander Essential Oil	high antibiofilm activity against *E. coli* biofilms contains 89.73% of terpenes (shown to have antimicrobial activity)	MIC: 1.6 μg/mL	[65]
	Pomegranate Extract	Antimicrobial activity against *E. coli * Inhibit formation of *E. coli * biofilms Complete eradication of *E. coli * biofilms	MIC: 250 μg/mL	[66]
Silver Nanoparticles (AgNPs)	AgNPs from AgNO_3_ and *Pandanus odorifer* leaf extract	anticancer potential by inhibiting migration of rat basophilic leukemic cells Antimicrobial activity against *E. coli* Reduces *E. coli* biofilm formation	MIC: 4 μg/Ml ~87% biofilm biomass reduction at 2 μg/mL	[67]
	AgNPs from *Gloriosa superba* aqueous leaf extract	Antibiofilm activity against *E. coli *	~44% biofilm thickness reduction	[68]
Upconverting nanoparticles (UCNPs)	Modified UCNPs (coupled with antibodies, covered with a shell surface made of TiO_2_ modified with d-amino acids)	Detect specific pathogens linked to CRC Antibiofilm activity through forming ROS and releasing d-amino acids	na.	[69,70,71]
Thiosalicylate Complexes	Thiosalicylate complexes of Zn(II) and Hg(II)	Complete inhibition of *E. coli * biofilms Antimicrobial activity against *E. coli * Anti-tumour actions against colon cancer cell line HCT 116	MIC: 0.227 μg/mL	[72]
Anti-rheumatic agent	Auranofin	Antimicrobial activity against *B. fragilis * Complete eradication of *B. fragilis* biofilms Inhibit formation of *B. fragilis* biofilms Reduction of *ompA* and *bmeB3* genes	MIC: 0.25 μg/mL	[73]
Probiotics	*Clostridium butyricum* NCTC 7423 Supernatant	Inhibit *B. fragilis* biofilms Eradicate *B. fragilis* biofilms Decrease metabolic activity Reduce *ompA* and *bmeB3 * Suppress extracellular nucleic acids and proteins	na.	[74]
	*Bifidobacterium* and *Lactobacillus*	Antibacterial activity against all *E. coli * Reduce the formation of biofilm of two multi-drug resistant *E. coli*	na.	[75]
Quorum sensing inhibitors	Quorum sensing inhibitors	Inhibits biofilm formations by: Inhibition of synthesis of signal moleculesSignal scrambling (degradation or sequestration) Interference with signal reception	na.	[76,77,78]

MIC—Minimum inhibitory concentration; na.—not available.
